# A novel mutation in *PCK2* gene causes primary angle-closure glaucoma

**DOI:** 10.18632/aging.203627

**Published:** 2021-10-14

**Authors:** Menghan Xu, Jin Yang, Jiayue Sun, Xuemei Xing, Zheng Liu, Tao Liu

**Affiliations:** 1 Department of Ophthalmology, 3201 Hospital, Xi’an Jiaotong University Health Science Center, Hanzhong, Shaanxi 723000, China; 2 The First People’s Hospital of Xianyang, Xianyang, Shaanxi 712000, China; 3 Department of Ophthalmology, Shaanxi Provincial People’s Hospital, Xi’an, Shaanxi 710068, China; 4 College of Medical Laboratory Science, Guilin Medical University, Guilin, Guangxi 541004, China; 5 Guihang Guiyang Hospital Affiliated to Zunyi Medical University, Guiyang, Guizhou 550000, China

**Keywords:** disease-causing mutation, primary angle closure glaucoma, *PCK2*, whole-genome sequencing, apoptosis

## Abstract

Primary angle-closure glaucoma (PACG) is an ophthalmic genetic disease characterized by direct contact between the iris and trabecular meshwork, resulting in an obstructed outflow of aqueous humor from the eye. However, it is unclear as to what role genetics plays in the development of PACG. The present study investigated the disease-causing mutation in a five-generation Chinese PACG family using whole-genome sequencing. A novel heterozygous missense mutation c.977C>T in *PCK2* gene was identified in five affected family members, but not in any unaffected and 86 unrelated healthy individuals. This nucleotide substitute is predicted to result in a proline to leucine substitution p.Pro326Leu. Furthermore, the function of this mutation was analyzed through various *in vitro* assays using the RGC-5 cell line. Our results demonstrate that the p.Pro326Leu mutation induces RGC-5 cell cycle arrest and apoptosis with a decreased BcL-XL. The increasing P53, P27, P21, AKT, and P-GSK3α were also detected in the cells transfected with c.977C>T mutation, suggesting that this mutation within *PCK2* gene cause PACG through impairment of AKT/GSK3α signaling pathway.

## INTRODUCTION

Primary angle-closure glaucoma (PACG) is the leading cause of irreversible blindness, which affected the worldwide population estimated to exceed 20 million in 2020 and over 30 million by 2040, with Asia accounting for 76% of PACG cases [1–2]. The prevalence of glaucoma varies significantly between different geographical regions and racial groups [3]. It is more common (60%) among women [4]. Based on recent reports, the prevalence of PACG in mainland China is estimated to be 1.4% (95% credible interval 1.0% to 1.7%) [5]. However, the prevalence of PACG among European derived populations aged 40 and over is only 0.4% (95% credible interval 0.3% to 0.5%) [6]. The pathogenesis of PACG is caused by direct contact between the iris and smooth muscle-like trabecular meshwork, which obstructs the aqueous humor pathway [7]. This obstruction prevents the exit of aqueous humor, thereby increasing the intraocular pressure, which is thought to degenerate the trabecular meshwork and damage the optic nerve [8]. The underlying causes of PACG remain unclear so far, but the genetic factors are well recognized in contributing to the pathogenesis of PACG [9].

During the past few years, genome-wide association studies have identified several PACG loci and genes, including GLC2A on chromosome 10q, PLEKHA7 on chromosome 11p, COL11A1 on chromosome 1p, MFRP on chromosome 11q [10]. SNPs in other candidate genes, including EPDR1, CHAT, GLIS3, FERMT2, DPM2-FAM102, also have a significant association with PACG [11]. Although numerous publications have pointed out these susceptibility genes that appear to contribute to developing PACG, strictly heritable disease-causing mutation has not been identified until Waseem et al. reported the first disease-causing mutation in *SPATA13* gene [12]. The discovery of strictly heritable disease-causing mutation can shed light on the molecular mechanisms of PACG.

In the present study, we recruited a five-generation Chinese PACG family. The strictly heritable disease-causing mutation of this family was identified by whole-genome sequencing with bioinformatics analysis. Additionally, this mutation was confirmed as a loss-of-function mutation by various experiments.

## RESULTS

### Clinical findings

**Figure 1:**
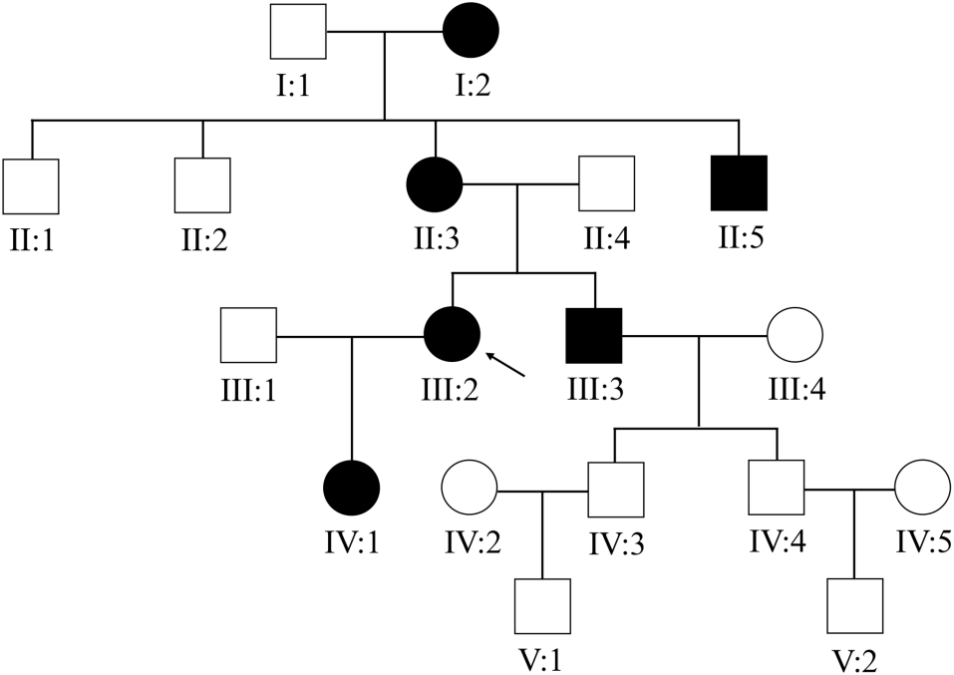
**The Chinese family with primary angle-closure glaucoma.** The pedigree of the five-generation Chinese PACG family is shown. Roman numerals indicate generations, and individuals within a generation are numbered from left to right. The proband (III:2) is denoted with an arrow.

**Figure 2:**
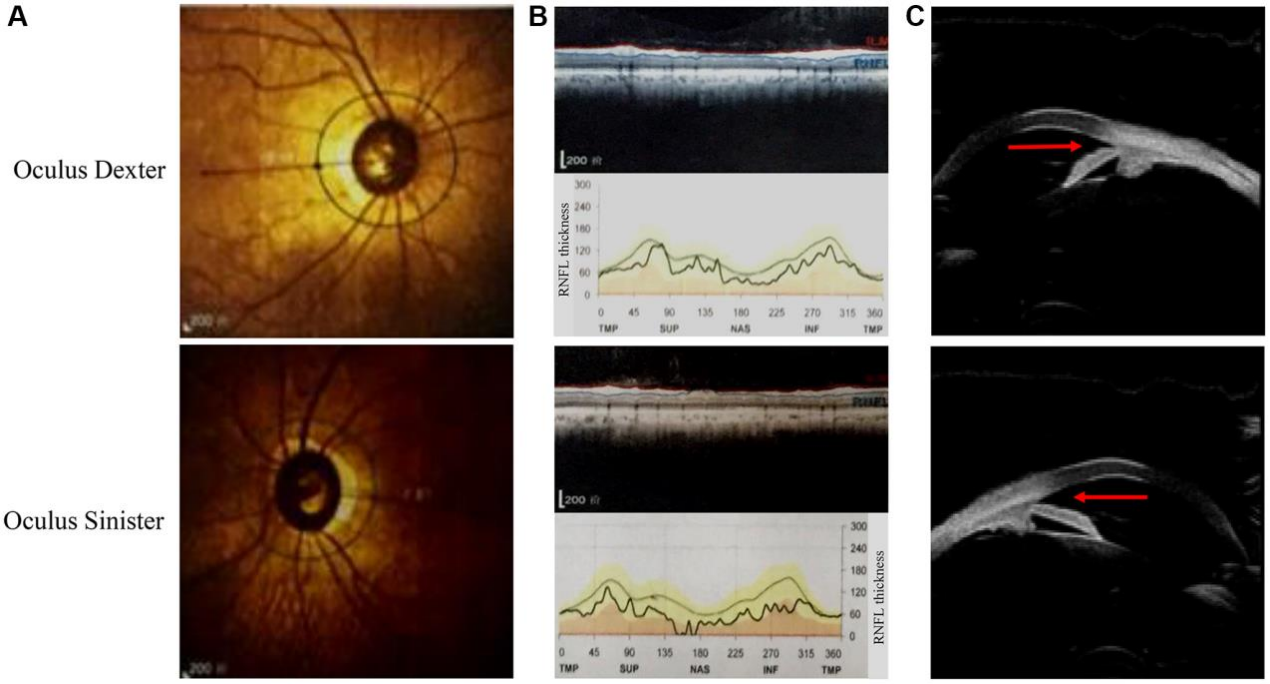
**Ophthalmic examination of the proband in this pedigree.** (**A**) The fundus angiography exam of the proband (III:2). (**B**) Retinal nerve fiber layer (RNFL) thickness is assessed by optical coherence tomography. (**C**) The results of ultrasonic biomicroscopy reveal relative pupillary block and a narrow-angle inlet.

This Chinese PACG family (Figure 1) comprises 18 members (10 males and 8 females). The proband III:2 at the age of 49 (black arrow) was enquired about a family history of glaucoma. The accuracy of family history information was confirmed by the corroboration of relatives attending the hospital. I:2 had been treated with conservative drug therapy for ocular hypertension for 35 years from age 60. On examination, all affected members were found to have the same phenotype as the proband III:2. The thickness of the optic nerve fiber layer in both eyes was significantly thinner. Direct contact of the iris root with the trabecular meshwork led to a closure of the anterior chamber angle (Figure 2). The proband’s mother II:3 and her uncle II:5 underwent surgery for glaucoma. This family has a strong history of glaucoma.

### Whole-genome sequencing and sanger sequencing

**Figure 3:**
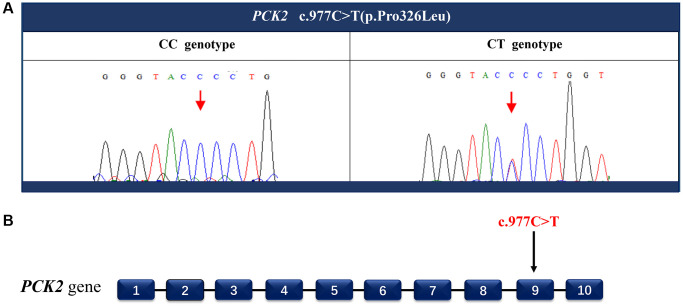
**Identify the disease-causing mutation in the PACG family.** A heterozygous missense mutation c.977C>T (**A**) located in exon 9 (**B**) of the *PCK2* gene was detected by Sanger sequencing. This nucleotide substitution results in a proline to leucine substitution p.Pro326Leu.

The whole-genome sequencing was performed to identify the disease-causing mutation. By using ClinPred, an efficient tool for identifying disease-relevant nonsynonymous variants, 18 variants (the scores >15) were identified as possible disease-causing mutations (Table 1). Sanger sequencing was applied to screen these potential pathogenic variants. A cytosine (C) to thymine (T) heterozygous missense mutation c.977C>T in phosphoenolpyruvate carboxykinase 2 (*PCK2*) gene was detected in all five affected family members (Figure 3), but not in any of the unaffected members and 86 unrelated individuals. The C to T nucleotide change at this locus is predicted to result in proline (P) to leucine (L) substitution p.Pro326Leu.

**Table 1:** The disease-causing mutation in candidate genes.

**Chr**	**Start**	**End**	**Ref**	**Alt**	**Gene**	**code**	**pro**	**ClinPred**	**CADD_Pred**
chr10	112541205	112541205	A	G	*RBM20*	c.A838G	p.K280E	0.610286035	16.53
chr10	32306144	32306144	C	A	*KIF5B*	c.G2688T	p.Q896H	0.991513669	21.8
chr12	58220831	58220831	C	G	*CTDSP2*	c.G302C	p.R101T	0.965011537	22.7
chr12	76453592	76453592	C	T	*NAP1L1*	c.G415A	p.E139K	0.888694346	19.66
chr13	32823672	32823672	A	C	*FRY*	c.A7018C	p.I2340L	0.893504195	33
chr14	24572375	24572375	C	T	*PCK2*	c.C977T	p.P326L	0.986453235	31
chr14	65259743	65259743	G	C	*SPTB*	c.C2638G	p.L880V	0.957589209	17.56
chr15	54838931	54838931	C	T	*UNC13C*	c.C5708T	p.S1903L	0.666001618	16.71
chr16	61761045	61761045	C	T	*CDH8*	c.G1489A	p.A497T	0.894139826	35
chr18	28914109	28914109	T	C	*DSG1*	c.T949C	p.W317R	0.998477757	18.39
chr1	227069657	227069657	C	T	*PSEN2*	c.C49T	p.R17W	0.887850463	20.4
chr1	227288725	227288725	T	G	*CDC42BPA*	c.A1974C	p.L658F	0.955309629	18.56
chr3	172737337	172737337	G	A	*SPATA16*	c.C787T	p.R263W	0.998399913	24.7
chr5	162880987	162880987	T	C	*NUDCD2*	c.A460G	p.N154D	0.776593506	16.95
chr5	96117517	96117517	G	A	*ERAP1*	c.C2327T	p.A776V	0.823308527	19.39
chr6	34008409	34008409	G	A	*GRM4*	c.C937T	p.P313S	0.982662201	21.9
chr7	75615259	75615259	C	T	*POR*	c.C1688T	p.T563M	0.837663054	19.31
chr8	144812605	144812605	C	T	*FAM83H*	c.G148A	p.E50K	0.966016054	23

Abbreviations: Chr: Chromone; Start: variant of start position; End: variant of end position; Ref: reference base; Alt: mutant base; pro: protein; ClinPred: score >0.5; CADD_pred: score>15.

### Influence of mutations on cell cycle and apoptosis

**Figure 4:**
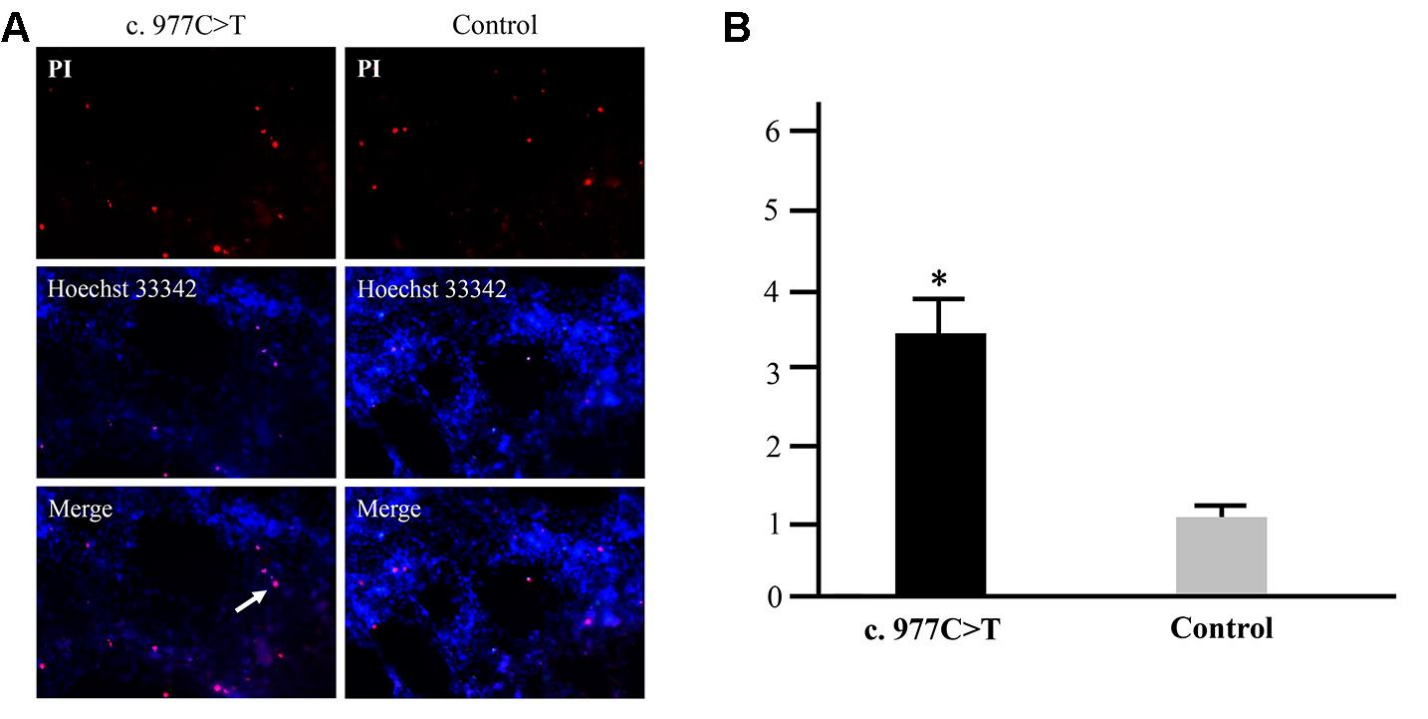
**Apoptosis analysis of the mutation p.Pro326Leu.** RGC-5 cells were transfected with wild-type *PCK2* or p.Pro326Leu mutation, respectively. Multiple apoptotic bodies (a, pink dots) were observed in the cells transferred with p.Pro326Leu mutation (**A**), and the apoptosis was further proved by flow cytometry (**B**). ^*^P < 0.05.

**Figure 5:**
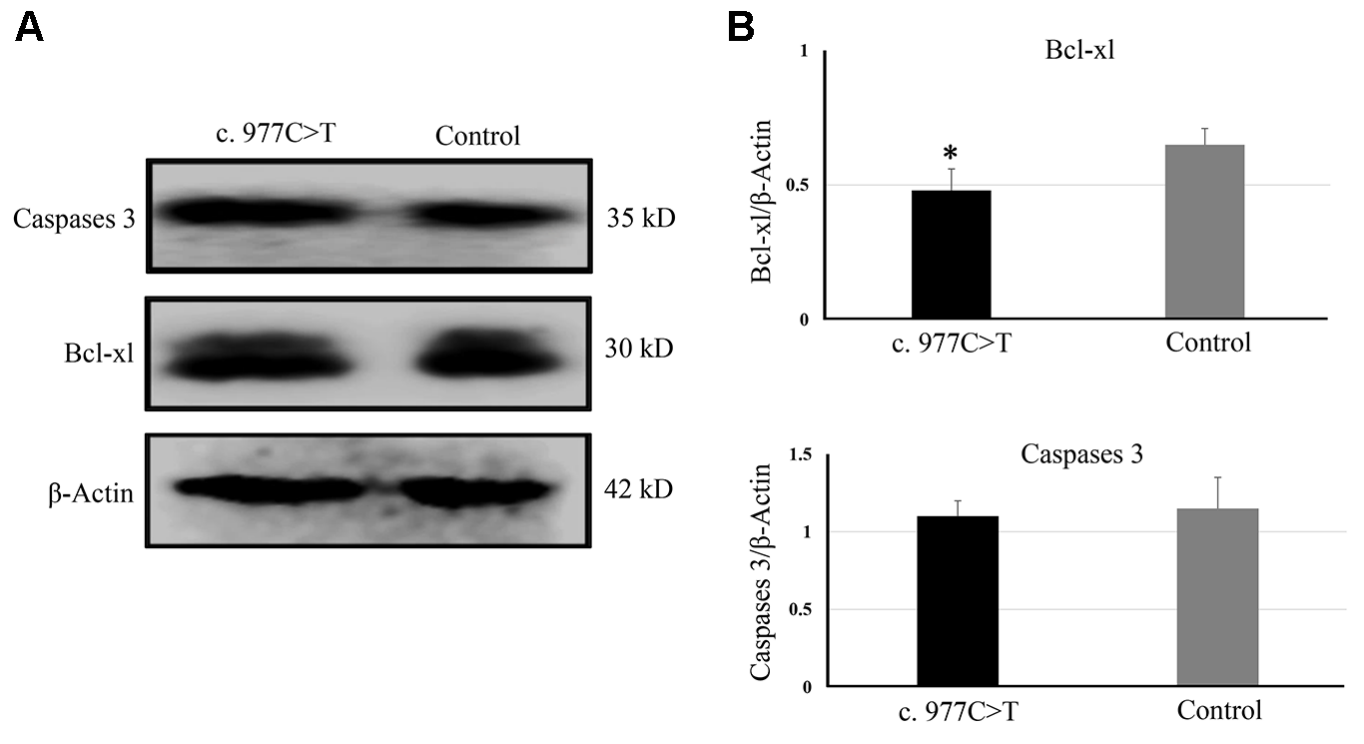
The expressions of genes related to apoptosis were determined by Western blotting (**A**) and quantitative analysis (**B**). ^*^P < 0.05.

**Figure 6:**
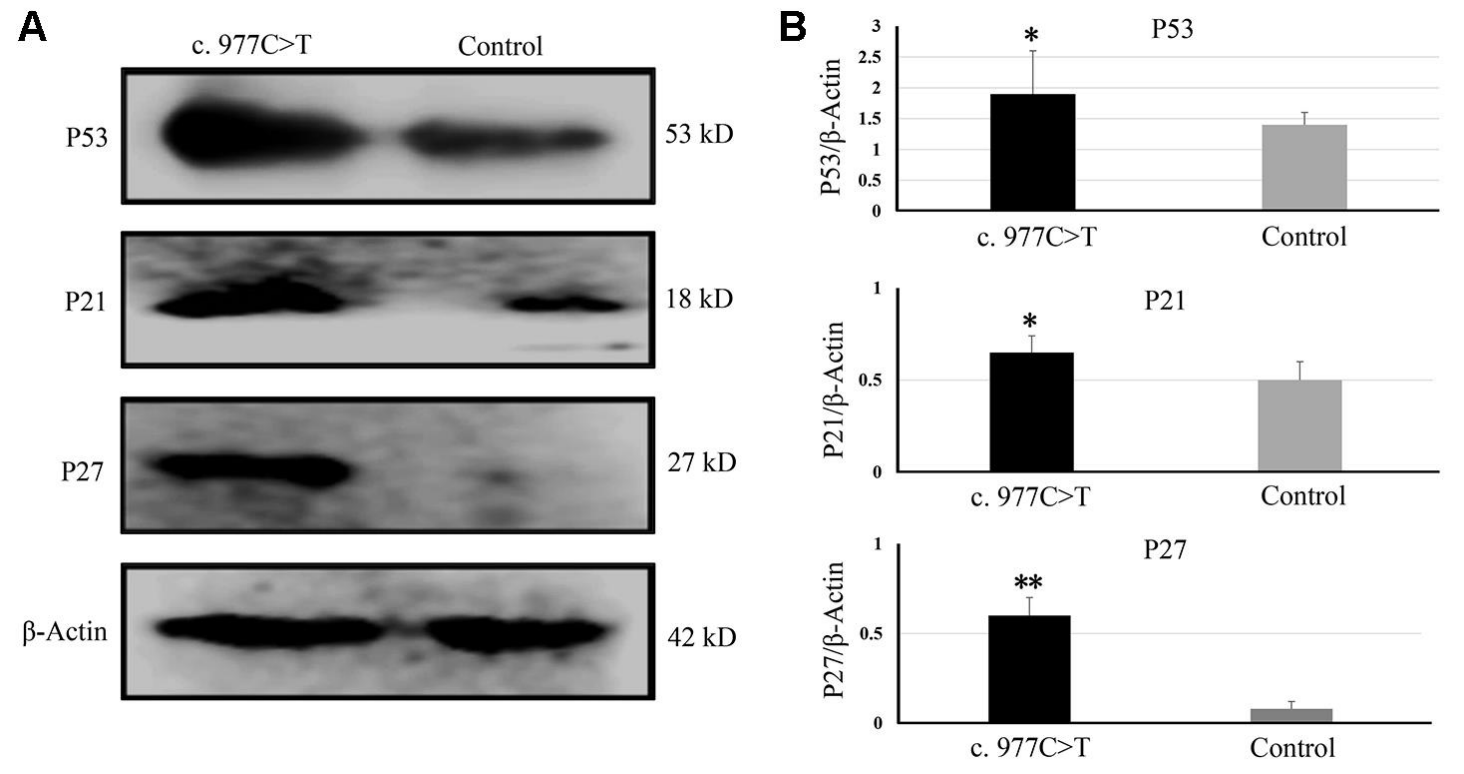
The expressions of genes related to the cell cycle and growth were determined by Western blotting (**A**) and quantitative analysis (**B**). ^*^P < 0.05; ^**^P < 0.01.

**Figure 7:**
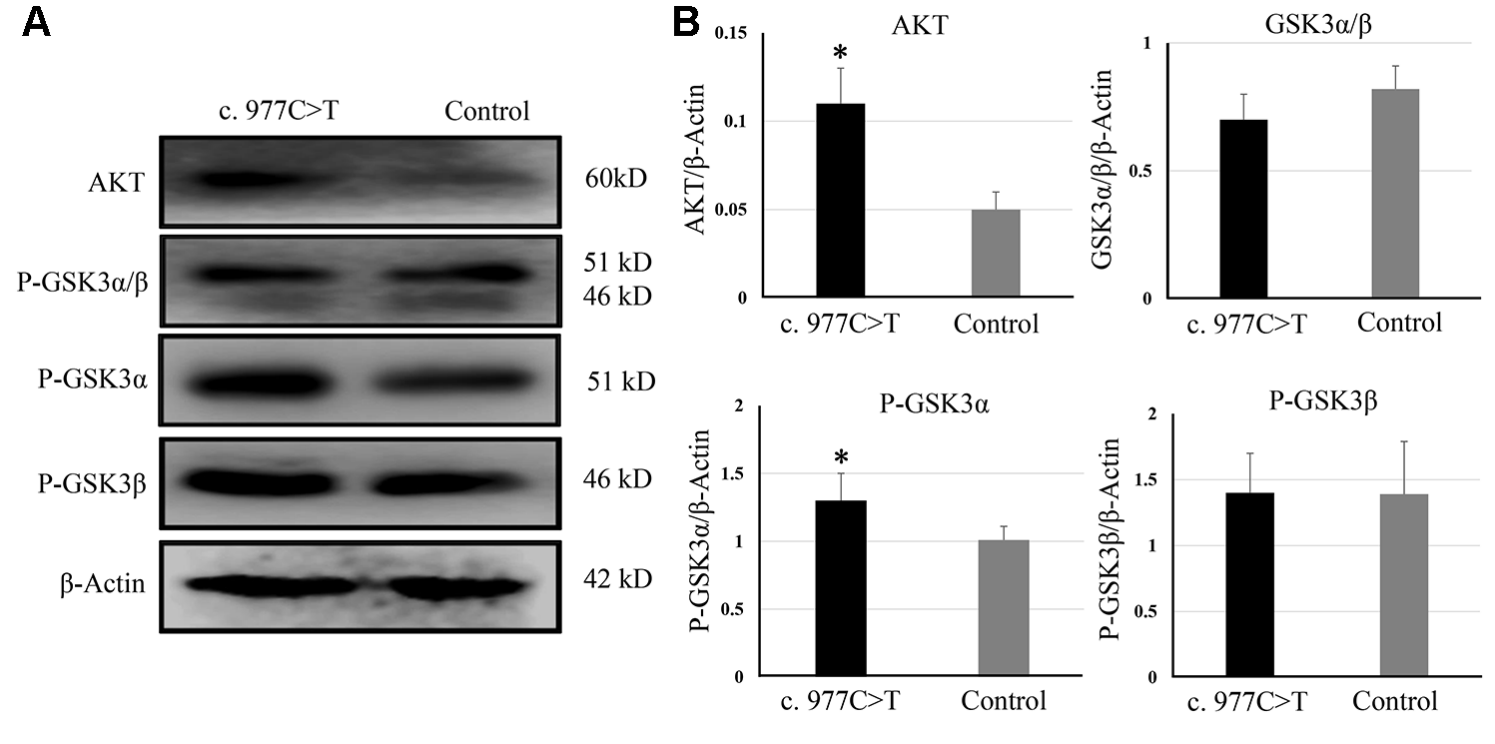
The expression of AKT, GSK3, P-GSK3α, and P-GSK3β were analyzed by Western blotting (**A**) and quantitative analysis (**B**). ^*^P < 0.05.

To further investigate the function of *PCK2* and determine the activity of the mutation on endogenous target genes, RGC-5 cells were stably transfected with constructs expressing *PCK2* with c.977C>T mutation. The mutation induces apoptosis of RGC-5, which was demonstrated by Hoechst 33342/PI double-fluorescent chromatin staining (Figure 4A) and flow cytometry (Figure 4B). In order to explore the mechanism of c.977C>T mutation-induced apoptosis, the levels of proteins associated with cell apoptosis were determined by Western blotting and quantitative analysis. A decreased BcL-XL was detected in RGC-5 cell lines transferred with c.977C>T mutation (Figure 5). To further investigate the effect of c.977C>T mutation on RGC-5 cells, the expression of P53, P21, P27, AKT, and GSK3 were measured. The elevated P53, P21, and P27 were observed in RGC-5 cell lines transferred with c.977C>T mutation (Figure 6). In addition, we found that the expression of AKT and phosphorylation of GSK3α were increased (Figure 7).

## DISCUSSION

The pathogenesis of PACG is complex. Many different factors affect the progression of the anterior chamber angle from narrow to angle-closure [13]. Among them, heritable factors play an important role in the etiology of PACG [14]. Therefore, singling out heritable factors contributing to the disease is crucial for unraveling the pathway of PACG development. In 2020, Waseem et al. identified the first disease-causing mutation c.1432_1440del in the *SPATA13* gene by analyzing a seven-generation British PACG family [12]. In the present study, we identified a novel disease-causing mutation c.977C>T in *PCK2* in a five-generation Chinese PACG family. PCK2 is a mitochondrial enzyme that catalyzes the conversion of oxaloacetate to phosphoenolpyruvate [15]. A previous study reported that the expression *PCK2* significantly was down-regulated in PACG [16], but the role of *PCK2* in the pathogenies and development of PACG remains largely unclear. The previous study demonstrated that the function of *PCK2* was to promote the growth of smooth muscle [17]. Hence, *PCK2* might involve the pathogenies of PACG by regulating the growth of smooth muscle-like trabecular meshwork, which plays a central role in the development of PACG. *PCK2* also is associated with lung cancer by promoting tumorigenesis through its gluconeogenic function [18]. Due to its gluconeogenic function, *PCK2* deficiency is expected to disrupt glucose homeostasis and result in hypoglycemia related-disorder [19]. The non-synonymous somatic variant in *PCK2* was identified in hepatocellular carcinoma tissue, suggesting that *PCK2* was associated with hepatocellular carcinoma [20].

To further explore the effect of mutation c.977C>T, the mutation was constructed into lentiviral vector pCD513B and transfected into RGC-5 cells. The c.977C>T mutation induces apoptosis of RGC-5, suggesting that c.977C>T mutation is a function-loss mutation. A decreased Bcl-xl indicates that c.977C>T mutation induces apoptosis by regulating the Bcl-xl induced-apoptosis pathways. Bcl-xL is a transmembrane molecule in the mitochondria, which acts as an anti-apoptotic protein by preventing mitochondrial release, leading to caspase activation and programmed cell apoptosis [21–22]. In addition, elevated P53, P21, and P27 were observed in RGC-5 cells transfected with c.977C>T mutation. P53 and its primary target P21 is linked with DNA damage to cell cycle arrest [23–24]. P27 is a negative regulator of cell proliferation and arrests the cell cycle by inhibiting cyclin-dependent kinases [25]. All of these proteins have been widely confirmed to be involved in cell cycle arrest to cell apoptosis.

In the present study, we also detected an increase of AKT and phosphorylation of GSK3α in RGC-5 cells containing c.977C>T mutation. Impairment of AKT/GSK3 signaling pathway in some eye disorders such as glaucoma, diabetic retinopathy, and retinitis pigmentosa have been reported during the past few years [26–27]. The function of AKT is to phosphorylate GSK3, which is a crucial regulator of differentiation, metabolism, and apoptosis [28]. The mechanism of the AKT/GSK3 signaling pathway involved in eye disorder is that increased GSK3 promotes inflammation and therefore may play a role in glaucoma and age-related macular degeneration activity under disease conditions [29–30]. Interestingly, we found that c.977C>T mutation only induced elevated phosphorylation of GSK3α, suggesting that GSK3α was the target of *PCK2* in the development of PACG. GSK3α and GSK3β are the two functional isoforms of GSK3, but whose functions remain ill-defined because of a lack of inhibitors that can distinguish between the two highly homologous isozymes [31–32]. The role of AKT and GSK3α in the pathogenesis of PACG needs to be further investigated.

In conclusion, we reported a novel heterozygous missense mutation in the *PCK2* gene causing PACG by analyzing a five-generation Chinese PACG family. Moreover, this mutation-induced RGC-5 cells apoptosis and elevated P53, P21, P27, AKT, and GSK3α, suggesting that this mutation c.977C>T within *PCK2* gene cause PACG through impairment of AKT/GSK3α signaling pathway. Identification of strictly heritable disease-causing mutation of PACG helps us to understand the glaucoma etiology.

## METHODS

### Ethical compliance

Written consent was obtained from all participants. The study was approved by the Hospital Research Ethics Committee (2017-002-SX), following the principles of the Declaration of Helsinki of 1975, as revised in 2000.

### Subjects and ophthalmic examination

All family members aged 18 years and older were invited to attend comprehensive ophthalmic examination, including fundus angiography, dark room gonioscopy, optical coherence tomography, ultrasonic biomicroscopy, visual field testing. The primary defining feature of cases was contact between the iris and trabecular meshwork identified on darkroom gonioscopy. PACG was diagnosed when there was evidence of iridotrabecular contact and glaucomatous damage to the optic nerve.

### Whole-genome sequencing and bioinformatics analysis

DNA was extracted from peripheral blood using Gentra PureGene kit (Qiagen, China). 86 random human DNAs were set as healthy control. Exome capture was performed with an Illumina HiSeq 2500 (Illumina Inc., CA, USA), and the raw sequencing reads were aligned by the BIOTECAN (Shanghai, China) using the Burrows-Wheeler Aligner. After removing duplicates from the sorted alignment using Picard, variants were called using the Genome Analysis Toolkit (GATK v3.70) pipeline. ANNOVAR was used to annotate SNPs and insertions/deletions. Filtrations of all identified variations were performed with data from public databases, including the database in human reference genome hg19 from UCSC, dbSNP, 1000 genomes project, and AVSIFT. After excluding common variants, retained variants were considered to be ‘novel’. Pathogenicity of the variants was evaluated ClinPred Prediction Tool. The criteria to filter candidate mutations were: 1. Located in exonic and splitting regions; 2. The variants frequency <0.05 in 1000g2012apr_all, esp6500si_all, and ExAC_ALL database; 3. The variants with CADD_Pred score >15 suggests potentially pathogenic variants. Direct Sanger sequencing was applied to confirm these potential pathogenic variants with the ABI3500 sequencer (Applied Biosystems, CA, USA). The primers for *PCK2* gene is 5′-CAGAGTTCTCCCCTGGTGAAT-3′ and 5′-AAAAGGAAGGTCTGGCTCAG-3′.

### Site-directed mutagenesis

Total mRNA was isolated from cultured cells using the Oligotex mRNA kit (QIAGEN, China) and transcribed into cDNA using the Transcriptor Reverse Transcriptase System (Roche, China). The coding regions of *PCK2* were amplified using high-fidelity DNA polymerase (New England Biolabs, China) and ligated into a lentiviral vector pCD513B. For site-directed mutagenesis, phosphorylated primers harboring the desired mutations were used to amplify the *PCK2* constructs with DNA polymerase and ligated with Taq DNA ligase. Sanger sequencing was used to verify all cloned inserts.

### Cell culture, virus packaging, and transfections

RGC-5 cell line and HEK293T were purchased from iCell Bioscience Inc, Shanghai, China. The cells were maintained in DMEM/F12 supplemented with 10% FBS and 1g/L glucose. HEK-293T cells were cultured in DMEM containing 10% FBS and 1% penicillin-streptomycin. All cells were grown in a 6-well plate at 1.2 × 10^6^ cells per well at 37°C in an atmosphere of 5% CO_2_. The packaging plasmids (pGag/Pol, pRev, and pVSV-G), pCD513B-PCK2 or pCD513B-PCK2-977C>T mutation were co-transfected into HEK 293T cells. Lentivirus-containing supernatants were collected and stored at −80°C.

### Flow cytometry

The effects of c.977C>T mutation on cell apoptosis were quantitatively analyzed by flow cytometry. RGC-5 cells in the logarithmic growth phase were inoculated into 6 cm dish and cultured for 48 hrs until the cells adhered to the wall. Following incubation, the cell culture medium was collected in a 10-mL centrifuge tube, and the cells were washed with PBS. Subsequently, the cells were centrifuged to remove the supernatant, re-mixed with binding buffer, and mixed with Annexin V-FITC and propidium iodide (PI) staining solution. After thorough mixing, the cells were incubated at room temperature in the dark for 15 min. PBS was added to the flow tube cell suspension, and a flow cytometry assay (Beckman Coulter) was performed.

### SDS electrophoresis and western blotting

Proteins were separated by SDS-polyacrylamide gel electrophoresis and transferred to a polyvinylidene difluoride membrane. Immunoreactive bands were visualized using the ECL Plus detection kit (HaiGene Biotechnology, Harbin, China). Anti-P53 and anti-P21 were purchased from Proteintech, Wuhan, China. Other antibodies (anti-P27, anti-P21, anti-Caspase 3, anti-Bcl-xl, anti-AKT, anti- P-GSK3α, anti- P-GSK3β, and anti-*β*-actin) were purchased from Sangon Biotech, Shanghai, China.

### Statistical analysis

Data are reported as the mean ± SD. Differences were considered statistically significant at *P* < 0.05. All statistical analyses were performed by using SPSS20.0 (SPSS Inc., Chicago, IL, USA).
